# Environmental stress and emotional reactivity: an exploratory experience sampling method study

**DOI:** 10.3389/fpsyt.2024.1375735

**Published:** 2024-04-26

**Authors:** Corine Sau Man Wong, Wai Chi Chan, Kristen Wing Yan Lo, Eric Yu Hai Chen, Linda Chiu Wa Lam

**Affiliations:** ^1^ School of Public Health, LKS Faculty of Medicine, The University of Hong Kong, Hong Kong, Hong Kong SAR, China; ^2^ Department of Psychiatry, School of Clinical Medicine, LKS Faculty of Medicine, The University of Hong Kong, Hong Kong, Hong Kong SAR, China; ^3^ Lee Kong Chian School of Medicine, Nanyang Technological University, Singapore, Singapore; ^4^ Department of Psychosis, Institute of Mental Health, Singapore, Singapore; ^5^ State Key Laboratory of Brain and Cognitive Sciences, The University of Hong Kong, Hong Kong, Hong Kong SAR, China; ^6^ Department of Psychiatry, Faculty of Medicine, The Chinese University of Hong Kong, Hong Kong, Hong Kong SAR, China

**Keywords:** environmental stress, affect, depression, momentary assessment, experience sampling

## Abstract

**Background:**

Previous studies have shown a relationship between environments and mental health. However, limited studies have investigated the impact of environment stress (ES) on emotional reactivity. Our study aimed to fill this gap by examining how daily ES affects momentary emotional reactivity using experience sampling method (ESM).

**Methods:**

Participants were randomly recruited from a prospective cohort study in Hong Kong to participate in a 7-day ESM study. The participants received eight electronic signals daily assessing their ES, positive affect (PA) and negative affect (NA). Participants were categorized into depressed group or control group based on Revised Clinical Interview Schedule. Psychometric properties of the ESM assessment were evaluated. Multilevel linear regression analyzes were conducted to examine the association of ES with PA, NA and the group status of the participants (cases versus controls).

**Results:**

A total of 15 participants with depression and 15 healthy controls were recruited, and 1307 momentary assessments were completed with a compliance rate of 77.8%. The depressed group demonstrated a significant increase in NA in response to ES, while the control group showed a decrease in PA. In addition, the depressed group reported a lower perception of control and interaction with their environment compared to the control group.

**Conclusion:**

Using ESM, a valid, reliable, and easy-to-use self-reporting tool, our findings provided valuable insights on the potential mechanisms underlying emotional responses to stressful environments.

## Introduction

With a large number of people living in cities, urban environments have become increasingly prevalent globally. Earlier studies have consistently highlighted the relationship between the environment and mood disorders (1). However, there is limited evidence on how the environment affects our everyday emotions. Emotions can fluctuate moment to moment in response to the stress from the surrounding environment, also known as environmental stress (ES) (2–4).

Understanding the connection between ES and emotional reactions is essential for developing effective interventions and promoting population mental health. Stress has been conceptualized as the individual’s subjective evaluation of the perceived stress of specific events and minor disturbances that occur as part of their daily routine (5). These daily stressors typically refer to the challenges of day-to-day living, ranging from common familial issues to workplace conflict and obstacles from the physical environment (6). ES is the perceived environmental-related stress in daily life, while immediate emotional reactions, such as positive affect (PA) and negative affect (NA), represent the response to these daily stressors (7). This complex interplay of emotional reactivity to ES has been explored using the experience sampling method (ESM), a structured self-reported technique designed to examine subjective momentary experiences in everyday life (8–10). In the early stages, ESM primarily used traditional pen-and-paper diaries or questionnaires. However, as technology advanced, electronic devices and smartphone applications have been integrated into momentary assessment (9, 11). Using electronic devices, participants are prompted at random intervals to fill out questionnaires capturing their current emotions, environment and activities. ESM offers the advantage of collecting data that concerns both the context (e.g., location and situation) and structure (e.g., the associations between environment and emotion) (12). These daily techniques provide a reliable means to study various aspects of a people’s life throughout the day (13). Utilizing ESM to collect real-time responses at multiple time points reduces recall bias and assessment error, improving ecological validity and reliability (14). It is widely used in studies on people with depression, psychotic disorder and substance abuse (15–17).

The impact of environment on emotional responses fluctuates among individuals. Studies have shown that stress-related emotions, in particular, sadness-depression, commonly arise from individual-environment interactions (18). Therefore, studying emotional reactions to environmental stress in people suffering from depression is important for understanding this dynamic. A recent systematic review found that the ESM, when deployed through electronic devices, effectively measures psychological outcomes such as mood and stress in adult patients with various physical conditions (11). Momentary affect has been largely correlated with the physical and social context of the environment. For instance, NA has been associated with being alone and at the hospital, whereas PA is often linked to environments and public locations crowded with people (19). Despite this, the application of smartphone-based ESM for studying urban stress and its psychometric properties is limited (20). Enhancing our understanding of environmental stressors and the dynamic change in emotional responses is particularly important considering the established relationship between the environment and mental health. By employing ESM, we can gain valuable insights into how people with depression respond to environmental stressors.

This exploratory study was designed to investigate the momentary emotional responses to daily ES using ESM. By collecting real-time data in naturalistic settings, we aimed to compare the effects of ES on individuals with and without depression. We hypothesized that ES would increase NA and decrease PA. As compared to healthy individuals, we hypothesized that participants with depression would exhibit greater emotional reactivity (both PA and NA), interact less with their environment, and perceive a lower sense of personal control over their surroundings.

## Materials and methods

### Study design and participants

This study used data from a 3-year longitudinal follow-up study of the Hong Kong Mental Morbidity Survey (HKMMS). The baseline HKMMS was a population-representative survey consisting of 5719 Chinese adults aged between 16 and 75 living in Hong Kong (21, 22). After 3 years, a follow-up study was conducted to investigate the long-term mental health outcomes. In this exploratory case-control study, invitations were randomly sent to a total of 45 participants (23 cases and 22 control subjects), out of which 33 of them (17 cases and 16 control subjects) agreed to participate in the study. One particular case was unable to be matched with control subjects due to the challenges encountered in finding a suitable match. A comparison between those who agreed to participate and those who declined was conducted, revealing no significant differences between the two groups in terms of age, gender, education level, marital status and employment status (p > 0.05). Participants with a score of 12 or higher on the Revised Clinical Interview Schedule (CIS-R) (23) were recruited as cases. Depression diagnosis was further ascertained using the Chinese-bilingual version of the Structured Clinical Interview for DSM-IV, Axis I, patient version (SCID) (24, 25). Control subjects were age-, gender- and education-matched to the cases, and had no lifetime history of mental disorders as defined by the SCID. Exclusion criteria included intellectual disability, dementia or significant cognitive impairment, lifetime history of psychotic disorders, major visual problem that affected perception of the surrounding environment, and inability to use a smartphone-based program. The study was carried out in accordance with the latest version of the Declaration of Helsinki. Ethical approval was obtained from the Institutional Review Board of the University of Hong Kong/Hospital Authority Hong Kong West Cluster (Reference No. UW15-304). Written informed consent was obtained from all participants at the time of recruitment.

### ESM procedure and assessments

Potential participants were contacted by phone to explain the aim and objective of this study. After obtaining participants’ consent, face-to-face interviews were conducted at their homes for an initial assessment and detailed explanation of the study procedures. Participants were given a smartphone (Samsung Galaxy J5) with the ESM application installed. The device was programmed to emit random electronic signals eight times a day between 10:00 am and 10:00 pm for seven consecutive days. At least one signal was scheduled within each 90-minute span using a random number table. The timing of the signals was designed to balance the participant burden while ensuring the collection of valid data at random timepoints throughout the day. On receiving each electronic signal, participants were prompted to complete a self-reported questionnaire within a 15-minute window. Any data submitted beyond this time were discarded. The same set of questionnaires was used throughout the study period, with each taking approximately 2 minutes to complete. The ESM procedure was explained to participants during a briefing session, and a practice trial was administrated prior to the initial data collection to ensure that the participants understood the instructions. Previous studies showed that the application of ESM was feasible and valid among both the general population (26) and people with depression (27).

At each signal, participants were first instructed to take a photograph of their current location, which was used to verify the surrounding environment. They were then asked to respond to a list of 20 ESM items (**Table 1**), most of which were rated on a 7-point Likert scale (1 = not at all; 7 = very much). Additional context-based questions regarding their location and social environment were answered by selecting from a list of options. Emotional states were assessed with four PA (items 2, 4, 6 and 9: happy, relaxed, satisfied and excited) and four NA (items 3, 5, 7 and 8: anxious, lonely, irritated and sad). PA and NA scores were derived from the average of their respective items. With reference to previous ESM studies on measuring activity- or event-related stress (5, 28, 29), we assessed ES by asking the extent to which participants currently experience discomfort with the environment. ES was considered any of the three self-reported items measuring arousal (item 10: I feel uncomfortable my current environment), frustration (item 11: I am frustrated with my current environment) and stress perceived from the surrounding environment (item 12: I would like to leave this place). ES score was calculated as the average of these three items. Interaction, personal control and social engagement in the environment were measured with items 13 to 18. A general statement of ‘my mood is affected by the surrounding environment right now’ was asked at the end of assessment. In addition to the ESM procedure, the CIS-R total scores were retrieved from the follow-up study in assessing psychological distress. The score was derived from 14 sections of non-psychotic symptoms, with higher scores indicating greater psychological distress (23, 30).

**Table 1:** List of ESM items.

No.	Type	Item
1	Snapshot	Please take a photograph of your current environment.
2	Emotional state	How happy do you feel right now?
3		How anxious do you feel right now?
4		How relaxed do you feel right now?
5		How lonely do you feel right now?
6		How satisfied do you feel right now?
7		How irritated do you feel right now?
8		How sad do you feel right now?
9		How excited do you feel right now?
10	Environmental stress	I feel uncomfortable with my current environment.
11		I am frustrated with my current environment.
12		I would like to leave this place.
13	Interaction	To what extent are you interacting with your environment right now?
14	Perceived control	To what extent do you have control of your environment right now?
15	Social context	Where are you right now? Home/Office/School/Transport/Social event/Work event/Others
16		How many people are with you right now? 0 1 2 3 4+
17		Who are you staying with right now? (check all that apply) Spouse/Family/Boyfriend or girlfriend/Colleague/Friend/Classmate/Stranger
18		How close do you feel with this person (these people)?
19	Mood by environment	My mood is affected by the surrounding environment right now.
20	Last signal effect	Since the last signal, the environment that I stayed was pleasurable.

### Data processing

During the 7-day assessment, each participant generated eight time-point entries per day, resulting in a maximum of 56 observations. Assuming full compliant from 30 participants, the dataset would comprise a total of 1680 observations. Data processing and cleaning were performed according to the data management guide for experience-sampling studies (31). Before data analysis, the ESM data were checked to identify any problematic entries. Specifically, we classified entries that took less than 10 seconds to complete (less than 0.5 seconds per item) as non-compliant responses, indicating the participant tapped the answers without considering the options. Furthermore, trials with over 90% of identical entries were considered as invalid and excluded from the analysis (32).

### Statistical analysis

The psychometric properties of the ESM assessment were first examined. The four PA items, four NA items and three ES items were assessed for internal consistency using Cronbach’s alpha coefficient. The convergent validity of the affect items was evaluated using correlational analysis of CIS-R total scores with PA and NA scores. The ESM data followed a hierarchical structure in which multiple momentary observations are nested within participants. Due to the highly correlated observations within each individual, multilinear regression modeling was employed to analyze ESM data variability at individual and group levels simultaneously (33). Prior research has proposed compliance rate cut-offs between 30% and 60% of prompts (28, 34–36). Given the limited exploration of momentary environmental stress, our study employed a higher compliance rate of 70% to effectively capture a comprehensive picture of participants’ daily experiences in this understudied area. Participants who completed less than 70% of the data points (fewer than 40 self-reports out of 56) were excluded from the analysis. Prior to analysis, the individual-level covariates (e.g., the ES) at different data points were centred around the individuals means (37). Multilevel linear regression analysis was first conducted with PA and NA as the dependent variables and ES as independent variables in the model. These models were then further adjusted for age, gender and the CIS-R total score. Additionally, in the separate ES-sensitivity models, ES, group status (0 = control, 1 = depressed) and their interaction term (ES × GROUP) were included as independent variables to examine whether the group status (healthy controls versus depressed participants) moderated emotional reactivity to the daily environment. The analysis of interaction and personal control with the environment between groups were conducted using same procedure. All statistical analyzes were carried out using SPSS Statistics version 20.0 (38).

## Results

### Participant characteristics

Seventeen participants with depression and sixteen healthy matched control subjects were recruited. Two individuals from the depression group and one from the control group failed to complete at least 40 entries (compliance rate ranging from 11% to 39%) and were excluded from the analyzes. The final sample consisted of 15 depressed cases and 15 control subjects. Women comprised two thirds of the sample. The mean age of the sample was 49.4 years (SD 8.9 years; range 31 to 66 years), and 80% of the participants had a secondary education or above. There were no significant differences between two groups regarding marital status (p = 0.256) or employment status (p = 0.143). The median CIS-R scores for the control subjects and depressed cases were 0 (range 0 to 7) and 23 (range 12 to 33), respectively. The sociodemographic and clinical characteristics of the sample are depicted in **Table 2**.

**Table 2:** Sociodemographic and clinical characteristics of the sample.

	Control group (n=15)	Depressed group (n=15)
Gender, n (%)		
Male	5 (33.3)	5 (33.3)
Female	10 (66.7)	10 (66.7)
Age group, n (%)		
31–45	4 (26.7)	5 (33.3)
46–60	8 (53.3)	8 (53.3)
≥61	3 (20.0)	2 (13.3)
**Age, mean (SD)**	49.73 (9.28)	49.07 (8.91)
Education level, n (%)		
Primary or below	3 (20.0)	3 (20.0)
Secondary	9 (60.0)	9 (60.0)
Tertiary or above	3 (20.0)	3 (20.0)
**Education year, mean (SD)**	12.78 (5.43)	11.90 (5.75)
Marital status, n (%)		
Married	11 (73.3)	8 (53.3)
Not married	4 (26.7)	7 (46.7)
Employment status, n (%)		
Working	9 (60.0)	5 (33.3)
Not working	6 (40.0)	10 (66.7)
CIS-R total score, median (range)	0 (0-7)	23 (12-33)

CIS-R, Revised Clinical Interview Schedule; SD, standard deviation.

**Table 3** presents the ESM characteristics of the sample. Of the 1680 signals sent, we received 1307 completed momentary assessments (675 from the control group and 635 from the depressed group). All of the participants completed at least 70% of the entries. The overall compliance rate was 77.8% (range 71.4% to 89.3%). The control group had a higher compliance rate (78.6%) than the depressed group (75%) (p = 0.008). Compared to the control group, the depressed group displayed a lower PA (p < 0.001), higher NA (p < 0.001) and increased ES (p < 0.001). The frequency distribution of the responses on PA, NA and ES are presented in **Supplementary Figures 1.1**, **1.2** and **1.3**, respectively. A comprehensive view of ESM responses on PA for 15 cases and 15 control subjects is shown in **Supplementary Figure 2**. Participants with depression reported spending more alone time (p < 0.001), had more time at home (p < 0.001) and were less engaged in social events (p < 0.001) than the control participants. They also indicated less impact of the environment on their mood (p < 0.001).

**Table 3:** ESM characteristics of the sample.

	Control group (n=15)	Depressed group (n=15)	p-value
**Total number of valid entries**	675	632	**––**
**Number of valid entries, median (range)** **^a^**	44 (41-50)	42 (40-46)	**<0.01**
**Compliance rate (%), median (range)** **^a^**	78.6 (73.2-89.3)	75.0 (71.4-82.1)	**<0.01**
**Positive affect, median (range)** **^a^**	4.5 (1.8-6.5)	2.0 (1.0-4.3)	**<0.001**
**Negative affect, median (range)** **^a^**	1.5 (1.0-4.0)	2.5 (1.0-4.8)	**<0.001**
**Environmental stress, median (range)** **^a^**	1.3 (1-4)	3 (1-7)	**<0.001**
Uncomfortable with the environment	1 (1-4)	3 (1-7)	**<0.01**
Frustrated with the environment	1 (1-4)	3 (1-7)	**<0.01**
Want to leave the current place	1 (1-4)	3 (1-7)	**<0.01**
**Social context, number of entries (%)** **^b^**			**<0.001**
Alone	119 (17.7)	173 (27.2)	
With others	553 (82.3)	462 (72.8)	
**Situation context, number of entries (%)** **^b^**			**<0.001**
Home	276 (41.1)	403 (63.5)	
Office	207 (30.8)	82 (12.9)	
At transport	103 (15.3)	107 (16.9)	
Social events	86 (12.8)	43 (6.8)	
**Mood affected by the environment, median (range)** **^a^**	3 (3-5)	3 (1-6)	**<0.001**

ESM, experience sampling method.

a Group differences by Mann-Whitney U test.

b Group differences by chi-square test.

### Psychometric properties of ESM assessment

The Cronbach’s alpha for momentary assessments was 0.96 for PA, 0.80 for NA and 0.98 for ES, indicating good internal consistency among the items in the three subscales (39). The correlation coefficient between PA and NA was moderately high (-0.64), suggesting a significant negative correlation between the two opposite affect items. Furthermore, a correlation of -0.68 of PA and a correlation of 0.51 of NA with the CIS-R total score suggested moderate to good convergent validity of the affect items.

### Association between ES and affects

Multilevel linear regression analysis revealed significant associations between ES and PA (B = -1.21; 95% CI -1.64 to -0.78; p < 0.001) and between ES and NA (B = 0.45; 95% CI 0.26 to 0.63; p < 0.001) in the full sample of 30 participants. These associations persisted after adjustment for age, gender and the CIS-R total score (**Table 4**). A significant interaction effect of ES × GROUP was found for both PA (B = 0.71; 95% CI 0.35 to 1.08; p < 0.001) and NA (B = -0.34; 95% CI -0.54 to -0.13; p = 0.003), indicating that the group modified the positive and negative emotional reactivity toward ES (**Table 5**). When stratified by group, ES was associated with a decreased PA for the control group (B = -1.77; 95% CI -2.39 to -1.16; p < 0.001), while no association was observed for depressed group (B = -0.12; 95% CI -0.80 to 0.57; p = 0.723). On the other hand, ES was associated with an increased NA for the depressed group (B = 0.35; 95% CI 0.03 to 0.68; p = 0.031), but this was not seen in the control group (B = 0.08; 95% CI -0.18 to 0.34; p = 0.527) (**Supplementary Table 1**). Moreover, participants with depression reported less interaction with the environment (B = -1.20; 95% CI -2.29 to -0.11; p = 0.032) and perceived less personal control over their environment compared to those in the control group (B = -1.37; 95% CI -2.52 to -0.22; p = 0.021) (**Supplementary Table 2**).

**Table 4:** Multilevel model estimates for PA and NA with ES as predictor (n=30).

	No. of observations	Mean (SD)		B (SE)	95% CI for B	p-value
		Control group (n=15)	Depressed group (n=15)			
**PA**						
Model 1^a^	1307	1.92 (0.9)	3.11 (0.9)	-1.21 (0.21)	-1.64 to -0.78	**<0.001**
Model 2^b^	1307	1.92 (0.9)	3.11 (0.9)	-1.25 (0.21)	-1.67 to -0.83	**<0.001**
**NA**						
Model 1^a^	1307	1.92 (0.9)	3.11 (0.9)	0.45 (0.09)	0.26 to 0.63	**<0.001**
Model 2^b^	1307	1.92 (0.9)	3.11 (0.9)	0.44 (0.09)	0.26 to 0.63	**<0.001**

CI, confidence interval; CIS-R, Revised Clinical Interview Schedule; ES, environmental stress; NA, negative affect; PA, positive affect; SD, standard deviation; SE, standard error.

a Multilevel model with PA and NA as dependent variables.

b Multilevel model with PA and NA as dependent variables, adjusted for age, gender and CIS-R total score.

**Table 5:** Multilevel model estimates for PA and NA with ES, GROUP and ES x GROUP as predictors (n=30).

	B (SE)	95% CI for B	p-value
PA			
Intercept^a^	5.38 (0.82)	3.71 to 7.05	**<0.001**
ES	-1.04 (0.17)	-1.38 to -0.70	**<0.001**
GROUP	-1.24 (0.51)	-2.28 to -0.19	**0.023**
ES x GROUP	0.71 (0.18)	0.35 to 1.08	**<0.001**
NA			
Intercept^a^	0.26 (0.29)	-0.33 to 0.84	0.383
ES	0.31 (0.10)	0.09 to 0.52	**0.008**
GROUP	0.05 (0.19)	-0.34 to 0.43	0.807
ES x GROUP	-0.34 (0.10)	-0.54 to -0.13	**0.003**

CI, confidence interval; CIS-R, Revised Clinical Interview Schedule; ES, environmental stress; GROUP, group status (0=control, 1=depressed); ES x GROUP, interaction term of environmental stress and group; NA, negative affect; PA, positive affect; SE, standard error.

a Multilevel model with PA and NA as dependent variables, adjusted for age, gender and CIS-R total score.

Bold values denote statistical significance at the p<0.05 level.

## Discussion

To our knowledge, our study is among the very few studies that employed ESM to investigate emotional responses to daily environmental stress. We recruited 15 participants with depression and 15 matched healthy controls, and 1307 momentary assessments were completed with a satisfactory compliance rate of 77.8%. The diary technique of ESM captures momentary emotions, events and environmental conditions through intensive and repeated self-report measures. By assessing participants’ perceptions of daily life conditions, ESM offers insights into underlying psychological processes (9, 40, 41). We specifically examined environment-related stress experience and emotional reactivity concurrently. The collection of real-time momentary experiences minimized retrospective bias, and therefore are able to reflect a more accurate relationship between the environment, stress and affective states at any given moment. Furthermore, our findings demonstrated excellent internal consistency for affect and ES assessments. The emotional states obtained through the ESM also correlated well with CIS-R measures, indicating good convergent validity of the affect items. In sum, ESM is a feasible, valid and reliable tool in assessing environmental factors and emotion in daily life.

### Environmental stress and emotional reactivity

Our study identified a significant relationship between ES and emotional reactivity. We noted that stress from the environment was associated with lower PA and higher NA, regardless of participants’ demographic characteristics or levels of psychological distress (42). However, emotional responses involving changes in various response systems such as feelings, perceptions, and behavior, may vary between healthy individuals and those with depression (43). We therefore examined whether participants’ health status (healthy versus depressed) altered emotional reactions to daily environment factors. We observed a notable increase in NA towards environmental stressors in the depressed group, while no such increase was found in control group. These results aligned with previous studies that individuals with depression tend to experience negative emotional reactions to daily stress across different contexts, such as events, activities and social interactions (28, 44, 45). For instance, Myin-Germeys et al. (28) examined emotional reactivity in different patient groups with severe mental illnesses and found that the group with major depressive disorder displayed greater stress-induced NA compared to healthy control subjects. In another ESM study of 279 twin pairs, Wichers et al. (46) suggested that the tendency to respond to daily life stressors with NA could be considered as a risk of depression. Moderately stressful events induced minimal effect to healthy people but greatly triggered NA in those with depression or a strong familial predisposition to depression. Our study built upon previous findings and provided further evidence that daily life stressors are not limited to specific events or activities, but also encompass the influence of physical environment.

### Environmental stress in depressed individuals

Negative emotional reactions to daily ES are a potential marker for depression, as supported by other observational studies linking stress induced by adverse environment to an increased depression risk (47, 48). This mechanism could be explained by stress sensitivity, where individuals with higher sensitivity to stress are more prone to developing affective symptoms (49). People with history of depression exhibit greater sensitivity to daily stresses, and even modest stressors can trigger NA. Our findings suggest that daily hassles in environment could heighten stress sensitivity in those with depression, thereby intensifying their negative reactions. Despite facing more ES, the depressed participants perceived their mood to be less affected by the environment. These findings suggest that people with depression may encounter stressful experience with limited awareness of how their mood is influenced by the environment.

### Environmental stress in healthy individuals

Notably, healthy control subjects in our study showed no changes in NA but exhibited a significant decrease in PA when exposed to environmental stressors. However, we could not observe this trend in depressed participants, which contradicts our hypothesis. Several factors could explain this discrepancy. First, the depressed group consistently had low PA levels throughout the study period, reflecting the pathological state of depression and made it difficult to detect emotional reactivity (28). Second, previous studies have shown that naturalistic acute stressors reduce hedonic capacity or the ability to experience pleasure in healthy subjects (50, 51). Perceived stress manifests as decreased PA rather than increased NA in non-clinical populations (52). Third, the healthy participants may have been more conscious of the surrounding environment than the depressed subjects. Many of them expressed concerns about the environment and its potential impact, which might have increased their sensitivity to emotional changes associated with their surroundings. Lastly, the effect of ES on affect may differ among individuals with different personality traits like agreeableness, neuroticism and extraversion (53, 54). Future studies should account for these factors while examining the affective response to ES.

### Environment and stress vulnerability

Our findings indicate a difference in emotional response to ES between people with depression and those without. Depressed individuals tend to manifest stress through NA, while healthy individuals exhibit stress through PA. These results suggest that the psychopathological pathway of depression may be associated with stress in urban environments. Ecological factors contribute to depression and may be influenced by other psychosocial mediators such as perceived control and interactions with physical environment. Individuals with a lower sense of control over their environment are less receptive to positive experiences and more sensitive to daily environmental stressors, may make them more susceptible to depression. In addition, our findings suggest that underlying vulnerability modifies how individuals respond to stressors in different situations. Intervention strategies should consider the environmental contextual factors and the psychosocial mechanism connecting everyday stressful environments to symptomatology.

### Strengths and limitations

This study has several strengths worthy of note. First, the methodological design using ESM offered not only strong ecological validity, but also good reliability, improved accuracy and reduced retrospective bias. By collecting real-time data on daily affect, environmental stress and other relevant information for a week, we assessed moment-to-moment experiences in participants’ everyday environment. To ensure a comprehensive understanding of these experiences, we placed great emphasis on achieving a high compliance rate among participants, ensuring a robust and diverse set of information for analysis. The ESM technique proves particularly beneficial for people with depression, who are frequently reported to have a negative retrospective bias (55, 56). Second, we focused on the stressful experiences the participants perceived from their environment instead of assessing the quality of the environment. This approach allowed us to unambiguously capture the adverse psychological effects (i.e., emotional reactivity) caused by suboptimal environments (57). Third, we incorporated multiple information sources, which enhanced the accuracy of the ESM measures. For example, participants were asked to provide photographs for location verification and CIS-R scores were retrieved from interview for between-subject measures. Finally, the use of semi-structured SCID interviews ensured adequate diagnostic verification and confirmation of symptom severity to support the participants’ eligibility.

Several methodological limitations must be addressed. Despite employing an advanced experience sampling method, this study was limited by its modest sample size and potentially limited generalizability of the findings. Further investigation with a larger sample size is warranted. In addition, a practice effect was commonly reported in ESM studies, especially among patients with major depressive disorder (27, 58). Although we did not observe a decrease in the response latencies over the course of the study (practice effect not supported), the depressed participants had a lower compliance rate compared to the controls, which could potentially contribute to a reduced statistical power. Nevertheless, the compliance rate of 75% in the depressed group was comparable with, and somewhat higher than the rates reported in previous studies (59, 60). Besides, it is important to note that while negatively appraised environments are not necessarily the same as stressful environments, there is often an overlap in daily life. Participants who are predisposed to high NA might report elevated ES. Similarly, the more intense an environment is, it tends to be perceived as negative (61). The response bias should be acknowledged. Another limitation was the self-reported nature of the ESM, which predominantly assessed the subjective affective states. Future studies should consider incorporating objective physiological measures, such as heart rate or cortisol level, to gain a more comprehensive understanding of the stress response to the environment. Finally, the concept of environmental stress and its associated contextual factors like location or social situation, have yet to be fully explored. Future research should explore the impact of ES on emotions in different contexts.

## Conclusion

In conclusion, this study highlighted the importance role of environment-related stress in regulating emotional responses. By utilizing ESM, a reliable and valid self-report tool, we observed significant variability of emotional reactions between depressed and healthy individuals. Importantly, exposure to environmental stressors led to a rise in NA for individuals with depression and a reduction in PA for the healthy participants. Furthermore, people with depression perceived less control over their environment and had less interaction with it. This study extended beyond previous studies by shedding light on the potential mechanisms underlying emotional reactions to stressful environments. The role of daily-environment stress-sensitivity fostered our understanding of the psychological mechanisms contributing to depression. Future investigation into this mechanism would help to develop ecological momentary interventions targeting resilience building towards daily environmental stressors and promoting effective self-management of emotions.

## Data availability statement

The raw data supporting the conclusions of this article will be made available by the authors, without undue reservation.

## Ethics statement

The studies involving humans were approved by Institutional Review Board of the University of Hong Kong/Hospital Authority Hong Kong West Cluster (Reference No. UW15-304). The studies were conducted in accordance with the local legislation and institutional requirements. The participants provided their written informed consent to participate in this study.

## Author contributions

CW: Conceptualization, Formal analysis, Methodology, Project administration, Visualization, Writing – original draft, Writing – review & editing. WC: Conceptualization, Funding acquisition, Supervision, Writing – review & editing. KL: Visualization, Writing – review & editing. EC: Funding acquisition, Supervision, Writing – review & editing. LL: Funding acquisition, Supervision, Writing – review & editing.
