# Supplementary material for: Environmental stress and emotional reactivity: an exploratory experience sampling method study
Source: Front Psychiatry. 2024 Apr 26;15:1375735. doi: 10.3389/fpsyt.2024.1375735 (PMC11106578; doi:10.3389/fpsyt.2024.1375735)
Supplement: Supplementary file 1 [file DataSheet_1.docx]

Supplementary Figure 1.1 Distribution of responses on a 7-point Likert scale of the PA items.

Supplementary Figure 1.2 Distribution of responses on a 7-point Likert scale of the NA items.

Supplementary Figure 1.3 Distribution of responses on a 7-point Likert scale of the ES items.

**Supplementary Figure 2** PA scores over daily ESM responses of 15 cases and 15 control subjects.


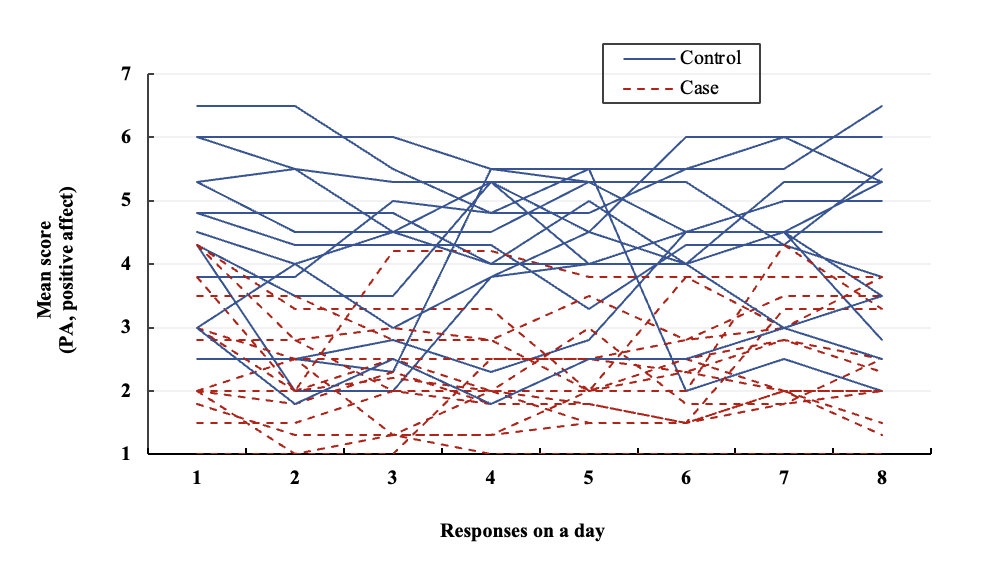


| Supplementary Table 1 Effect of ES on PA and NA, stratified by group. | | | | | | | |
| --- | --- | --- | --- | --- | --- | --- | --- |
|  | **Control group (n=15)^a^** | | |  | **Depress group (n=15)^a^** | | |
|  | **B (SE)** | **95% CI for B** | **p-value** |  | **B (SE)** | **95% CI for B** | **p-value** |
| PA | -1.77 (0.29) | -2.39 to -1.16 | **<0.001** |  | -0.12 (0.32) | -0.80 to 0.57 | 0.723 |
| NA | 0.08 (0.12) | -0.18 to 0.34 | 0.527 |  | 0.35 (0.16) | 0.03 to 0.68 | **0.031** |
| CI, confidence interval; CIS-R, Revised Clinical Interview Schedule; ES, environmental stress; NA, negative affect; PA, positive affect; SE, standard error. | | | | | | | |
| ^a^ Multilevel model with PA and NA as dependent variables, adjusted for age, gender and CIS-R total score. | | | | | | | |

| **Supplementary Table 2** Interaction with environment and perceived personal control over environment (n=30). | | | |
| --- | --- | --- | --- |
|  | **B (SE)** | **95% CI for B** | **p-value** |
| **Interaction with environment^a^** |  |  |  |
| Intercept | 3.20 (0.78) | 1.60 to 4.81 | **<0.001** |
| GROUP (controls as reference group) | -1.20 (0.53) | -2.29 to -0.11 | **0.032** |
| **Perceived personal control over environment^b^** |  |  |  |
| Intercept | 2.98 (0.83) | 1.29 to 4.67 | **0.001** |
| GROUP (controls as reference group) | -1.37 (0.56) | -2.52 to -0.22 | **0.021** |
| CIS-R, Revised Clinical Interview Schedule; ESM, experience sampling method; GROUP, group status. | | | |
| ^a^ Multilevel model with ESM Item No. 13 as dependent variable, adjusted for age, gender and CIS-R total score. | | | |
| ^b^ Multilevel model with ESM Item No. 14 as dependent variable, adjusted for age, gender and CIS-R total score. | | | |
